# Coma in fatal adult human malaria is not caused by cerebral oedema

**DOI:** 10.1186/1475-2875-10-267

**Published:** 2011-09-17

**Authors:** Isabelle M Medana, Nicholas PJ Day, Navakanit Sachanonta, Nguyen TH Mai, Arjen M Dondorp, Emsri Pongponratn, Tran T Hien, Nicholas J White, Gareth DH Turner

**Affiliations:** 1 Nuffield Department of Clinical Laboratory Sciences, The John Radcliffe Hospital, University of Oxford, Oxford, UK; 2 Nuffield Department of Clinical Medicine, The John Radcliffe Hospital, University of Oxford, Oxford, UK; 3 Mahidol-Oxford Research Unit, Faculty of Tropical Medicine, Mahidol University, Bangkok, Thailand; 4 Department of Tropical Pathology, Faculty of Tropical Medicine, Mahidol University, 3rd Floor, 60th Anniversary Chalermprakiat Building, 420/6 Rajvithi Road, Bangkok 10400, Thailand; 5 Hospital for Tropical Diseases, Ho Chi Minh City, Viet Nam

## Abstract

**Background:**

The role of brain oedema in the pathophysiology of cerebral malaria is controversial. Coma associated with severe *Plasmodium falciparum *malaria is multifactorial, but associated with histological evidence of parasitized erythrocyte sequestration and resultant microvascular congestion in cerebral vessels. To determine whether these changes cause breakdown of the blood-brain barrier and resultant perivascular or parenchymal cerebral oedema, histology, immunohistochemistry and image analysis were used to define the prevalence of histological patterns of oedema and the expression of specific molecular pathways involved in water balance in the brain in adults with fatal falciparum malaria.

**Methods:**

The brains of 20 adult Vietnamese patients who died of severe malaria were examined for evidence of disrupted vascular integrity. Immunohistochemistry and image analysis was performed on brainstem sections for activation of the vascular endothelial growth factor (VEGF) receptor 2 and expression of the aquaporin 4 (AQP4) water channel protein. Fibrinogen immunostaining was assessed as evidence of blood-brain barrier leakage and perivascular oedema formation. Correlations were performed with clinical, biochemical and neuropathological parameters of severe malaria infection.

**Results:**

The presence of oedema, plasma protein leakage and evidence of VEGF signalling were heterogeneous in fatal falciparum malaria and did not correlate with pre-mortem coma. Differences in vascular integrity were observed between brain regions with the greatest prevalence of disruption in the brainstem, compared to the cortex or midbrain. There was a statistically non-significant trend towards higher AQP4 staining in the brainstem of cases that presented with coma (*P *= .02).

**Conclusions:**

Histological evidence of cerebral oedema or immunohistochemical evidence of localised loss of vascular integrity did not correlate with the occurrence of pre-mortem coma in adults with fatal falciparum malaria. Enhanced expression of AQP4 water channels in the brainstem may, therefore, reflect a mix of both neuropathological or attempted neuroprotective responses to oedema formation.

## Background

Cerebral malaria (CM) is a diffuse but potentially reversible encephalopathy, caused by infection with the protozoan parasite *Plasmodium falciparum*. CM presents clinically with decreased consciousness, seizures and coma. The treated mortality rate is high (15-30%), and there may be long-term neurological and developmental sequelae in survivors, particularly young children. However, no major neurological deficit is detectable in the majority of survivors, suggesting that the processes leading to coma may be rapidly and potentially completely reversible [1,2].

The genesis of coma in CM is multifactorial. The microvascular pathology of human CM is unique, and caused by *P. falciparum*-parasitized red blood cells (PRBC) adhering to vascular endothelium and other erythrocytes, causing microvascular obstruction, eliciting endothelial activation and signalling, blood brain barrier leakage and a range of both pathogenic and protective responses [3,4]. This process is termed sequestration. It is quantitatively greater in the cerebral microvasculature of patients with CM than in those who die without preceding coma [5,6].

Although microvascular obstruction is the pathological hallmark of cerebral malaria the degree of cerebral hypoxia alone does not satisfactorily explain either the coma of CM or the excellent recovery in the majority of survivors. Whether there is a link between sequestration and disruption to the integrity of the microvasculature causing consequent cerebral oedema, and whether oedema itself is a major cause of coma or death in CM, remains unproven. Post-mortem studies in south-east Asian adults show variable degrees of brain swelling, and whilst this is more common in African paediatric patients, both groups rarely show tentorial or brainstem displacement resulting from mass effects [7]. Some individuals die with preceding clinical symptoms suggesting brainstem herniation, but others with these signs may recover. Imaging studies *in vivo *demonstrate a variable degree of brain swelling [8-12]. The intravascular biomass of sequestered parasitized erythrocytes and secondary microvascular dilation and congestion undoubtedly contributes considerably to the increases in cerebral volume seen on imaging and the brain weights measured at autopsy [5], and there is often macroscopic evidence of brain swelling.

In this study, using post-mortem brain tissue from 20 Vietnamese patients who died of severe falciparum malaria, changes in vascular integrity were characterized by examining neuropathological evidence of cerebral oedema. This was done by assessing macroscopic evidence for brain swelling (such as brain weight or brainstem herniation) at autopsy and histological quantitation of patterns of oedema in the brain, including mild localised forms that may not have had a major impact on the overall brain water content and swelling, but could have altered the extracellular milieu and hence affected neuronal function. In addition, differences in the prevalence of oedema between different brain regions were examined and correlated with separate measures of changes in vascular integrity, to identify different patterns of oedema that may reflect different aetiologies. The contribution of severe systemic disease to changes in the vasculature and oedema was assessed by correlating neuropathological data with clinical and biochemical parameters in the patients pre-mortem.

Subsequently, a substudy was performed on the brainstem of 20 fatal malaria cases, because this was the region with greatest evidence of disruption to vascular integrity, and investigated using immunohistochemistry for the activation of the vascular endothelial growth factor (VEGF) signalling pathway via VEGF receptor 2 (phosphorylated KDR), that is a known potent activator of vascular permeability in the brain [13,14]. As the water content of the brain can also be modulated independently of changes to the BBB through differential expression of water channels, the expression of aquaporin 4 (AQP4) protein was examined with immunohistochemistry. AQP4 is the most abundant aquaporin in the brain belonging to a family of small integral water channel proteins, as this has been implicated in brain oedema associated with various neurological conditions [15].

## Methods

### Case selection and brain fixation

Brain specimens were taken at autopsy from adult patients who had died of severe falciparum malaria on the Malaria Ward, Hospital for Tropical Diseases, Ho Chi Minh City, Vietnam, as described previously [16]. Brains were removed whole, and fixed suspended in 10% neutral buffered formalin for a minimum of six weeks, and then dissected with a formal brain cut for sampling of different areas. These patients were divided into two groups: CM and non-CM. CM was defined according to World Health Organization guidelines [17], on the basis of a Glasgow coma score of less than 11, other causes of unconsciousness having been excluded (e.g. hypoglycaemia, meningitis or other encephalopathy) by clinical, biochemical and cerebrospinal fluid examination. Non-CM patients were those who died from severe malaria without coma. These patients had a range of clinical features typical of other vital organ system complications (see Table 1 for more details). Protocols for tissue sampling, storage and use were approved by the Ethical and Scientific Committee of the Hospital for Tropical Diseases, Vietnam, OXTREC 029-02, COREC (C01.002) and the Human tissue authority (HTA, UK) license number 12217.

**Table 1:** Malaria patient data

*Patient no*	*Study*	*Drug*	*Age (yrs)*	*Sex*	*Time to death (hours)*	*GCS*	*Seizures*	*CSF opening pressure* *(mmHg)*	*Additional Clinical* *Complications*
CM1	1	A	28	M	70	9	Yes	Normal (12)	J, A, ARF, HP, HG, Ac
CM2	1	Q	51	F	3	5	No	Normal (7)	A, ARF, HG, Ac
CM3	1	A	43	M	36	5	No	High (29)	PO, S, HP, Ac
CM4	1	A	28	M	4	9	No	ND	ARF, HP, S, Ac
CM5	1	A	26	M	171	8	Yes	High (19)	J, A, ARF, S
CM6	1,2	Q	36	F	52	6	No	High (20)	J, A, ARF, HG, S, Ac
CM7	1,2	A	22	M	38.5	7	No	Normal (9.5)	J, HP, HG, S, Ac
CM8	2	A	69	M	336	8	No	ND	J, A, ARF, HG, S
CM9	2	Q	34	M	6.6	8	No	Normal (10)	J, A, ARF, HG, PO, S, Ac
CM10	2	Q	36	M	20.66	8	Yes	High (23)	J, ARF, HP, HG, S
CM11	2	Q	30	M	36	7	No	Normal (17)	J, A, ARF, S, PO, Ac
CM12	2	Q	29	M	9	6	No	High (24)	PO, S, Ac
CM13	2	Q	63	M	16	10	No	Normal (9)	A, HG, PO, S
CM14	2	Q	44	M	39	7	No	High (21)	J, ARF, HG
CM15	2	A	44	F	24	7	Yes	Normal (17)	A, S
NCM1	1	A	26	M	45	11	No	Normal (12)	A, HG
NCM2	1	Q	27	M	185	15	No	ND	J, A, ARF, HP, HG, S, PO, Ac
NCM3	1	Q	34	M	4	15	No	ND	J, A, ARF, HP, Ac
NCM4	1	A	38	M	394	15	No	ND	HP, J, A, ARF, S, Ac
NCM5	1	A	33	M	24	14	No	ND	J, ARF, A, S, Ac
NCM6	1	Q	28	M	44	11	No	ND	J, ARF, A, HP
NCM7	1	A	25	M	151	15	No	ND	J, ARF, A, HG, S
NCM8	1	Q	32	M	18	15	No	ND	J, ARF, S
NCM9	1,2	Q	22	M	6.33	14	No	ND	J, HP, S, PO, Ac
NCM10	1,2	Q	63	M	50	11	No	High (21.5)	J, A, ARF, PO, S
NCM11	1,2	Q	43	F	94.5	15	No	ND	J, A, ARF, HP, HG, S
NCM12	1,2	Q	25	M	124	15	No	ND	J, A, ARF, HG
NCM13	2	Q	22	M	6	11	No	Normal (14)	J, A, ARF, HP, S, Ac
NCM14	2	Q	22	F	27.3	12	No	ND	J, ARF, HG, HP, S
NCM15	2	Q	54	M	35	11	Yes	Normal	J, A, ARF, S, Ac
NCM16	2	A	56	F	4.66	13	No	ND	J, ARF, S, PO, Ac
NCM17	1,2	Q	24	M	113	14	No	ND	J, A, ARF, S
NCM18	2	A	50	F	264	15	No	High (19)	J, A, ARF, S

Abbreviations: GCS, Admission Glasgow Coma Score; CSF Cerebrospinal Fluid; Q, quinine; A, artemether; CM, cerebral malaria; NCM, non-cerebral severe malaria; J, jaundice; A, anaemia; ARF, acute renal failure; HP, hyperparasitaemia; HG, hypoglycaemia; S, shock; PO, pulmonary oedema; Ac, Metabolic Acidosis. High CSF pressure > 18 mmHg, ND, not determined. Study 1, Histopathology; Study 2, Immunohistochemistry on brainstem sections

This project used two different sets of patient tissues (see Table 1). The first study (study 1) focused on histological types of oedema, plasma protein leakage and frank vascular damage in the form of haemorrhages across three different brain areas of the cortex, diencephalon and brainstem of each case (n = 20). The second study (study 2) focused on the VEGF and AQP4 pathways of oedema formation in the brainstem as these contain many of the key nuclei regulating cardiorespiratory function, malfunction of which might cause respiratory arrest and because the initial assessment showed this to be the area showing most oedema (n = 20, some of which overlapped the oedema cases, making the total number of cases examined 33, as shown in Table 1). There was no significant difference in the frequency of the occurrence of severe complications between the two study groups.

The prevalence of different histological patterns of oedema (per patient per brain area) was determined to examine whether histological types of oedema differed between different brain regions or patient groups. The relationship between prevalence of histological oedema and other neuropathological features of CM such as sequestration, microvascular congestion and haemorrhages was determined. In addition, the patterns of oedema were correlated with other clinical features of severe malaria infection, such as acute renal failure or seizures, by correlation with clinical and biochemical data on these patients. Quantitative and semi-quantitative immunohistochemical analysis provided insight into plasma protein leakage reflecting vasogenic oedema, and specific pathways involved in water balance in the brain at the terminal stage of severe malaria.

### Light microscopy and assessment of oedema and haemorrhages

**Figure 1:**
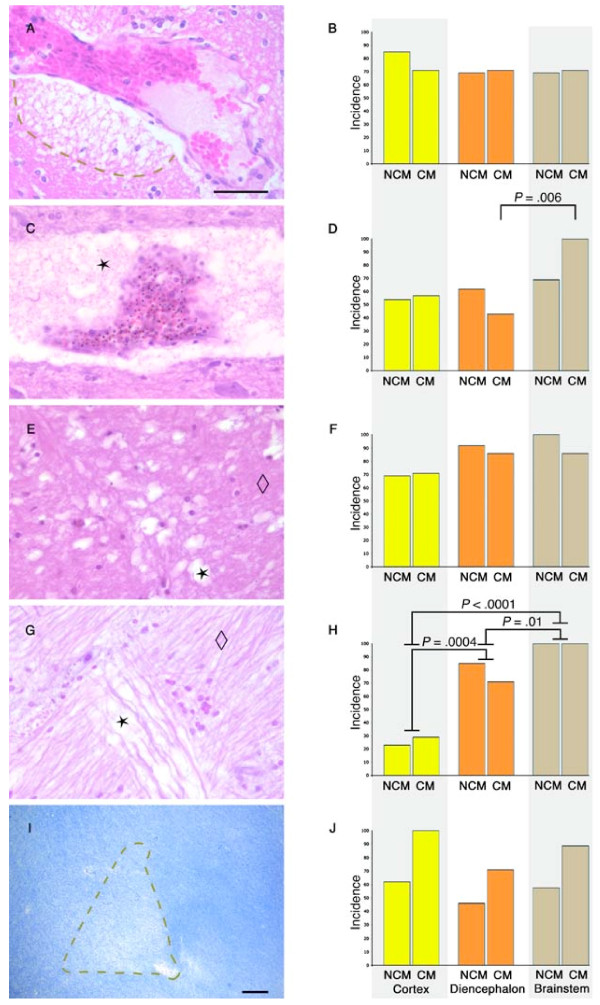
**Histological types of oedema in severe malaria**. **Left column: **Examples of the different histological types of oedema observed in the brain of fatal severe malaria in adults visualised with haematoxylin and eosin (A, C, E, G) or Luxol fast blue cresyl violet (LBCV, I). **Right column: **graphs indicating the incidence (% of cases) of the different histological types of oedema in cortex (yellow), diencephalon (orange) and brainstem (brown) of cerebral malaria (CM) and non-CM cases. No error bars are given as the bar chart represents the percentage of cases with or without a categorical histological feature. **A**. Rarefaction of the perivascular space characterized by separation of the compact parenchyma by fluid filled spaces (indicated by dashed line). **B**. There was no significant difference in the prevalence of perivascular rarefaction between CM and non-CM cases or between different brain regions. **C-D**. Perivascular pools of proteinaceous material indicated with a star (C). There was a greater prevalence in the brainstem compared with the diencephalon (*P *= .006) of CM cases (D). **E-F**. Vacuolar parenchymal oedema characterized by small isolated spaces (star) compare with compact parenchyma (diamond); (E). There was no difference between CM and non-CM cases or between different brain regions (F). **G-H**. Oedema between fibres of white matter tracts (star) compare with compact fibre tract (diamond); (G). There was a hierarchy of prevalence: brainstem > diencephalon > cortex. However, there was no difference between CM and non-CM cases (H). **I-J**. Decreased staining intensity of LBCV as a result of increased fluid-filled spaces between myelin fibres. Note myelin pallor in area indicated by dashed line radiating from the vessel in the bottom right corner (I). There was no significant difference between CM and non-CM cases or between different brain regions (J). Scale bar in A = 50 μm (for images A-G); scale bar in I = 100 μm.

**Figure 2:**
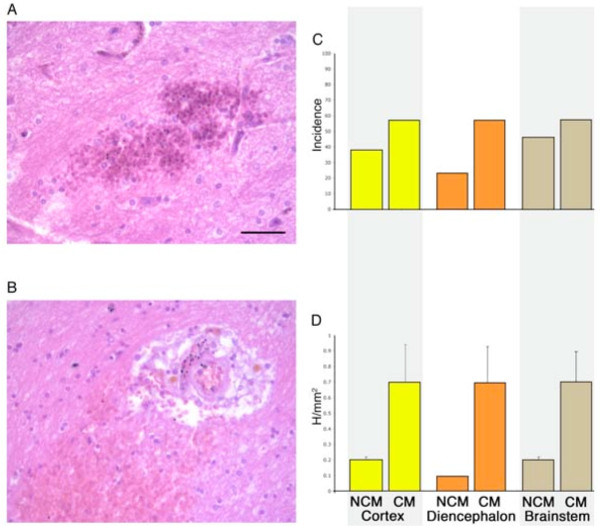
**Assessment of frank vascular damage in the form of haemorrhages**. **A-B**. Haemorrhages associated with small (A) and large (B) vessels visualized with haematoxylin and eosin stain. Scale bar = 50 μm. **C-D**. Quantitation of haemorrhages in severe malaria expressed as either the incidence of any haemorrhages in a case (% cases; C) or number of haemorrhages per mm^2 ^(error bars indicate SEM; D) in cortex (yellow), diencephalon (orange) and brainstem (brown). There was no significant difference in the prevalence or number of haemorrhages between CM and non-CM cases or between different brain regions.

Light microscopy was performed on paraffin embedded sections of formalin-fixed material from cortex, diencephalon and brainstem of each case. Sections were stained with Haematoxylin and Eosin for examination of oedema and haemorrhage and luxol fast blue cresyl violet (LBCV) histochemical staining to detect myelin pallor. Sections were examined under a 40× field of a Nikon Optophot microscopy by two independent observers (IM and GT). The presence or absence of a histological feature was noted for each section. The prevalence of the following types of oedema were assessed: perivascular rarefaction (Figure 1A); perivascular pools of proteinaceous material (Figure 1C); vacuolar appearance of the parenchyma (Figure 1E); loose or sieve-like appearance of myelinated axonal fibre tracts (Figure 1G) and pallor of myelin staining with Luxol Blue Cresyl Violet (Figure 1I). The prevalence of haemorrhages (simple punctate or ring haemorrhages) indicating frank vascular disruption was also noted and the number/mm^2 ^was calculated (Figure 2).

### Immunohistochemistry

Immunohistochemistry was performed on brainstem sections using the following antibodies or anti-sera: phosphorylated KDR (monoclonal, 1:5), kind gift, of H. Turley, NDCLS, University of Oxford [18]; AQP4 (1:200, polyclonal rabbit, Autogen Bioclear), glial fibrillary acidic protein (GFAP, clone: GF2, culture supernatant, kind gift of J. Cordell, OxFabs, NDCLS, Oxford), and fibrinogen (rabbit antiserum, DAKO). Paraffin sections were dewaxed, rehydrated and then underwent microwave antigen retrieval. Bound antibody was visualized using the EnVision HRP/AP kit (DAKO, UK) or the Novolink Polymer detection system (Leica Biosystems Newcastle Ltd, UK).

### Quantitation of immunostaining using digital image analysis

AQP4, GFAP, pKDR and fibrinogen immunolabelling was quantitated using a modified version of a semi-automated method used previously to quantitate ß-amyloid precursor protein (ß -APP) with tissue sections from severe malaria cases [19]. Briefly, tissue sections were digitized using the EverSmart Pro II flat bed scanner (CreoScitex, Canada). Regions of low, medium and high levels of immunolabelling were selected by density thresholding of grey scale converted images using SigmaScan Pro5 image analysis software (SYSTAT, San Jose, CA). Thresholds were selected by comparison of the staining intensity on brain sections taken from control cases, which autopsy brain tissues taken from patients dying in the UK from a variety of non-neurological and neurological causes (as detailed previously in [5] and [19]) as guides for setting the computerized low and high thresholds respectively. Thresholds were kept constant between cases for each marker. The total area of the tissue sections was calculated. The amount of marker load was expressed as the area of tissue covered by staining divided by the total area of the section in μm^2^.

### Semi-quantitation of cellular and vessel associated fibrinogen immunostaining

**Figure 3:**
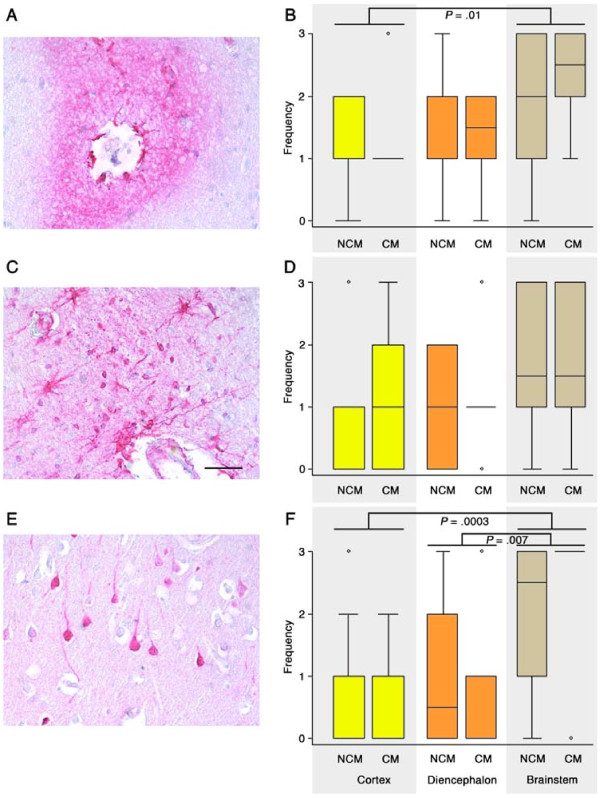
**Extravasation of fibrinogen reflecting vasogenic oedema**. **Left column: **Different fibrinogen staining patterns in the brain parenchyma: diffuse (A), glial- (C) or neuronal-associated (E). Combinations of these patterns could be observed on the same section. **Right column: **The frequency of the different fibrinogen staining patterns were evaluated using the semi-quantitative scoring system: no staining (0), < 1% cells or vessels staining/grade 1, 1 - 10% cells or vessels staining/grade 2, > 10% cells or vessels staining/grade 3 in addition to measurement of total fibrinogen load (see Figures 4 & 5). The diffuse pattern of staining may reflect a more recent leak compared to cellular uptake of fibrinogen that may indicate an older lesion or cell injury. There was no significant difference in the frequency of fibrinogen extravasation between CM and non-CM cases (B, D, F) but there were differences between different brain regions. Greater numbers of vessels showed diffuse fibrinogen leakage in the brainstem compared with the cortex of severe malaria cases (B). Neuronal uptake of fibrinogen was more frequently observed in the brainstem compared with diencephalon or cortex (F).

There were several different fibrinogen staining patterns in the brainstem parenchyma: diffuse, glial- or neuronal-associated and combinations of these patterns (Figure 3). The degree of fibrinogen immunolabelling associated with vessels (small and large), glia and neurons was assessed using the following semi-quantitative scale: no staining (-), < 1% cells or vessels staining/grade 1 (+), 1 - 10% cells or vessels staining/grade 2 (++), > 10% cells or vessels staining/grade 3 (+++). This was deemed necessary in addition to the quantitative digital measurement of total fibrinogen load since a diffuse pattern of staining may reflect a more recent leak compared to cellular uptake of fibrinogen that may indicate an older lesion. Furthermore, it has been observed that plasma proteins can be taken up into neurons with histological evidence of hypoxic injury [20].

### Statistical analysis

Statistical analysis was conducted using SPSS 13 (Chicago, USA). *P *< .01 was regarded as significant. The amount of immunostaining for the markers described above was correlated with the neuropathological evidence of parasitized erythrocyte sequestration (defined as the percentage of vessels containing parasitized erythrocytes) and vascular congestion in the same section. Clinicopathological correlations were conducted with cerebrospinal fluid (CSF) opening pressure, and the presence of other complications of severe malaria as defined by WHO criteria [17]. These included anaemia (haematocrit < 20% plus parasitaemia > 100,000 μl), haemodynamic shock (systolic blood pressure < 80 mmHg), pulmonary oedema, hypoglycaemia (plasma glucose < 2.2 mmol/L), admission peripheral parasitaemia, jaundice (bilirubin > 2.5 mg/dL), hyperlactataemia as a measure of metabolic acidosis (plasma lactate > 5 mmol/L) and acute renal failure (plasma creatinine > 3 mg/dL). Data were analyzed using appropriate non-parametric tests (Kruskal-Wallis test, Spearman rank correlation, and Fisher's exact test) depending on the continuous or categorical nature of the data.

## Results

### Histological types of oedema in severe malaria

Rarefaction of perivascular spaces is characterized by separation of the compact parenchyma by fluid filled spaces (Figure 1A). This contrasts with the complete separation of the vessel from the surrounding pia that can be a vessel shrinkage artefact resulting from fixation. There was no significant difference in the presence of perivascular rarefaction between CM and non-CM cases, no difference between different brain regions (Figure 1B) and no correlations with clinical, biochemical or histological parameters of severe malaria

The prevalence of the accumulation of proteinaceous material in perivascular spaces (Figure 1C) was greater in the brainstem compared with the diencephalon (*P *= .006, Figure 1D). No differences were seen between brain areas in non-CM cases (Figure 1D). The prevalence of proteinaceous material in perivascular spaces in the cortex was greater in severe malaria cases with longer time from admission until death (*P *= .01). The mean time to death from admission was 114.94 h (44.27 - 185.61: 95% CI) in cases with this pattern of oedema in perivascular spaces compared with 40.3 h (0 - 83.94) in those without.

Vacuolar parenchymal oedema was characterized by small isolated spaces that often coalesced (Figure 1E). However, there was no difference between CM and non-CM cases (Figure 1F). Cases with sections showing cortical, vacuolar parenchymal oedema had significantly greater wet brain weights at autopsy compared with cases without this type of cortical oedema [Mean 1370 g (SD 1323 - 1417 g) versus 1250 g (1149 - 1351); *P *= .01]. There were no other correlations with clinical, biochemical or histological parameters of severe malaria.

Oedema between the fibres of white matter tracts was observed in the brainstem of all severe malaria cases (Figure 1G). The prevalence of this pattern of oedema was significantly higher in the brainstem than the cortex (*P *< .0001) and diencephalon (*P *= .01), and the diencephalon was significantly more in cortex (*P *= .0004) (Figure 1H). However, there was no difference between CM and non-CM cases. There were no other correlations with clinical, biochemical or histological parameters of severe malaria infection.

Myelin pallor was characterized by decreased staining intensity of LBCV resulting from decreased myelin density because of increased spaces between myelin fibres, filled with fluid rather than representing frank demyelination (Figure 1I). All CM cases showed myelin pallor in the cortex. However, there was no significant difference in the prevalence of myelin pallor between CM and non-CM cases, no difference between different brain regions (Figure 1J) and no correlations with clinical, biochemical or histological parameters of severe malaria.

### Assessment of frank vascular damage in the form of haemorrhages

Microscopic haemorrhages were a common finding in severe malaria cases associated with both small and larger calibre vessels (Figure 2A and 2B) but were less frequent than histological signs of oedema in most brain areas (compare Figure 1 and 2). Overall more CM cases than NCM showed haemorrhages (Figure 2C). However there was no significant difference in the prevalence of haemorrhages or the number of haemorrhages per mm^2 ^between CM and non-CM cases because of the wide variation in absolute numbers of haemorrhages between cases. No difference between the number of haemorrhages in different brain regions was seen (Figure 2D) and no correlations with clinical, biochemical or histological parameters of severe malaria infection, such as sequestration or vascular congestion. In particular no significant relationship was found between the number of haemorrhages in any of the three brain regions and time to death (Cortex p = 0.51; Diencephalon p = 0.21; Brainstem p = 0.22).

### Assessment of plasma protein leakage using fibrinogen immunohistochemistry

Immunohistochemistry for fibrinogen was performed as another method to assess vascular integrity. The BBB usually excludes molecules < 500 Da so extravasation of fibrinogen, a large molecular weight plasma protein (340 kDa) into the tissue parenchyma reflects vasogenic oedema. Different staining patterns may reflect different age of leakage, with diffuse staining being more recent and cellular uptake into glia or neurons reflecting an older leak or cell damage allowing increased uptake into the cells. It should be noted that fibrinogen staining would not indicate increased permeability to smaller molecules < 340Da that may contribute to overall water content.

There were several fibrinogen-staining patterns including: diffuse (Figure 3A-B), glial (Figure 3C-D) or neuronal-associated (Figure 3E-F) as well as combinations of these patterns. However, there was no difference between CM and non-CM cases for any of the fibrinogen staining patterns (Figure 3B, D and 3F). There were differences between different brain areas. Greater numbers of vessels showed diffuse fibrinogen leakage, and neuronal uptake of fibrinogen was more frequently observed in the brainstem compared with the cortex of severe malaria cases (*P *= .01, .0003, respectively; Figure 3B, F). There was no difference between the diencephalon and brainstem with the exception of neuronal uptake that was more frequently observed in the brainstem (*P *= .007; Figure 3F).

The perivascular staining patterns were also correlated with vessel size. It was noted whether the sections showed perivascular vascular fibrinogen staining of small vessels only (capillaries and post-capillary venules), larger vessels only (arterioles and venules) or staining of both small and larger vessels. It was also noted whether the perivascular staining was diffuse or cell-associated. There was a greater prevalence of sections with only small vessels staining in the cortex compared with the diencephalon (*P *= .004). Conversely, there was significantly more perivascular fibrinogen staining in total in the brainstem compared with the cortex (*P *= .005).

Sections with histological evidence of oedema did not show more perivascular staining of fibrinogen or glial or neuronal uptake of fibrinogen compared with sections without histological evidence of oedema. Correlating clinical or biochemical parameters with patterns of fibrinogen staining showed that patients with jaundice had greater glial uptake of fibrinogen (*P *= .01) and less diffuse perivascular staining of small vessels (*P *= .01) in the brainstem at the time of death compared with cases without jaundice. Patients with acute renal failure showed greater numbers of diffuse and cell-associated perivascular fibrinogen leakage in the brainstem compared with cases without acute renal failure. No significant relationship was found between the amount of fibrinogen staining and the time to death (p = 0.30).

### Quantitative image analysis in the brainstem

The brainstem was chosen for quantitative image analysis of fibrinogen leakage and examination of specific molecular pathways that are known to be involved in oedema formation and water balance in the brain. The brainstem was selected as the area for the immunohistochemical arm of this study because loss of vascular integrity was more prevalent in this area in the microscopic study, compared to the midbrain and cortex. Respiratory arrest is a common terminal event in cerebral malaria. The reticular activating system, involved in the maintenance of consciousness, lies within the core of the pons and medulla. Damage to the cardio-respiratory centres in the medulla will lead to death.

#### Fibrinogen

**Figure 4:**
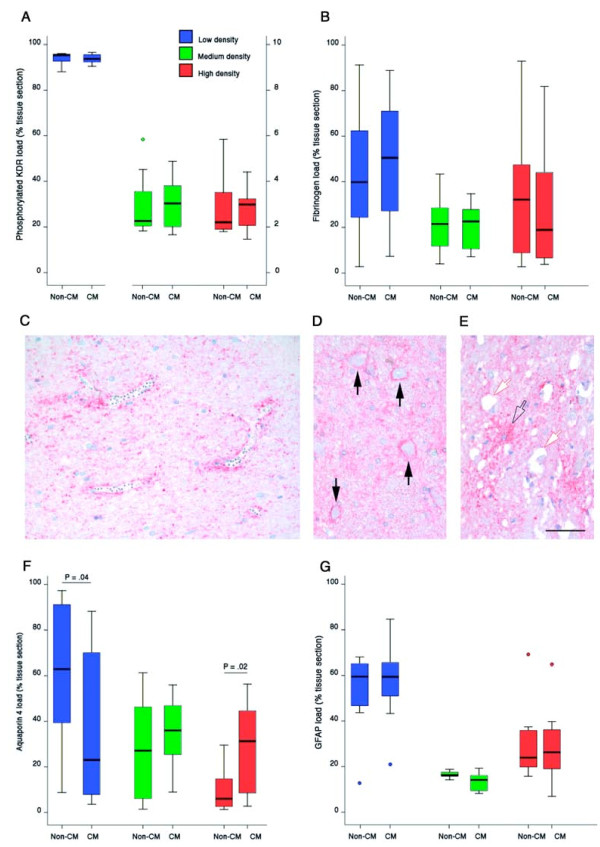
**Quantitation of immunolabelling for pKDR, fibrinogen, AQP4 and GFAP and patterns of AQP4 immunolabelling in post-mortem brainstem sections**. **A-B, F-G**. Quantitation of low, medium and high levels of immunolabelling for pKDR (A), Fibrinogen (B), AQP4 (F), and GFAP (G). Comparisons are made between severe malaria patients with cerebral malaria (CM) and those that died from other severe complications of malarial disease (non-CM). Data are presented as box and whisker plots showing median, lower quartile, the upper quartile and outliers. **C**. Enhanced perivascular labelling for AQP4 around small vessels containing parasites. **D**. Perineuronal AQP4 labelling in a CM patient (black arrows) in an area without histological evidence of oedema. **E**. AQP4 labelled astrocyte (empty arrow) in an area showing vacuolar oedema (white arrows). Scale bar = 50 μm (C-E).

**Figure 5:**
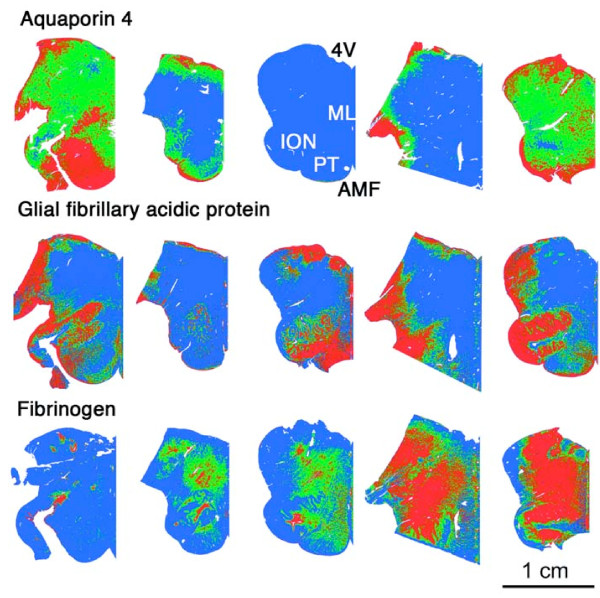
**Quantitation of AQP4, GFAP and fibrinogen using digital image analysis**. Brain maps showing low (blue), medium (green) and high (red) levels of immunolabelling for Aquaporin 4 (top row), Glial fibrillary acidic protein (GFAP; middle row) and Fibrinogen (bottom row). Maps of pKDR are not shown since all images appear almost entirely blue at this magnification. Each image represents half a transverse section of brainstem separated from the other half for presentation purposes at the 4^th ^ventricle (4V) to the anterior median fissure (AMF). For orientation, other histological features have been labelled on the middle image, first row: medial lemniscus (ML), inferior olivary nucleus (IOL) and pyramidal tracts (PT). Each column shows serial sections from the same patient staining for the different markers. Tissue sections were digitized, regions of low, medium and high expression were selected by density thresholding and areas were calculated and summated using SigmaScan Pro5.

There was considerable heterogeneity in the portion of sections displaying low, medium and high-density fibrinogen immunolabelling within the CM and non-CM groups (see Figure 4B &5). There were no correlations with clinical, biochemical or histological parameters of severe malaria infection with the exception of admission parasite count. High-density fibrinogen staining was significantly negatively correlated with admission parasite count (r = -.705, *P *= .001) whereas low density fibrinogen labelling was correlated with admission parasite count (r = .582, *P *= .007). There were no statistical correlations between sequestration or vascular congestion in the brain, the prevalence of histological types of oedema or brain weight at the time of death.

#### VEGF signalling

To determine whether VEGF signalling influences vascular integrity in severe malaria an activation state-dependent antibody was utilized. Only cells/vessels with receptors exposed to sufficient levels of ligand to trigger autophosphorylation and subsequent signalling were visualized. Using phosphorylated KDR antibody immunolabelling, VEGF signalling in severe malaria was demonstrated in vascular endothelial cells, glial cells and neurones. The portion of the brainstem sections showing low, medium and high density of pKDR immunolabelling is shown in Figure 4A. No significant differences between non-CM and CM cases were found. There were no correlations with clinical, biochemical or histological parameters of severe malaria infection. These findings are in agreement with a previous study, where the frequency of VEGF expression associated with the vasculature, glia and neurons within several brain regions was investigated [21]. There were also no statistical correlations/associations with sequestration or congestion levels in the brain, the prevalence of histological types of oedema or brain weight at the time of death.

#### Aquaporin 4

Strong AQP4 staining could be found in a punctate fashion in a peri- or paravascular location on astrocyte foot processes and perivascular glial cells around vessels of all calibre, with and without sequestered malaria parasites (Figure 4C). Enhanced staining was also found juxtaposed to neurons (Figure 4D) and within the parenchyma (Figure 4E).

Variable levels of AQP4 immunolabelling were observed in the brainstem of individual cases with severe malaria (Figures 4F and Figure 5) although highest levels were associated with the subpial regions. There was a non-significant trend for the portion of brainstem containing strong AQP4 staining to be higher in CM cases compared with non-CM cases (*P *= .02). CM cases showed high density AQP4 immunolabelling in 31.3% (Interquartile range [iqr] 8.59-44.59) of the brainstem section compared with 6.0% (iqr 2.65-14.71) in non-CM sections (*P *= .02). AQP4 is expressed predominantly by astrocytes, although discordant immunostaining between APQ4 and the astrocyte-specific marker glial fibrillary acidic protein (GFAP) is commonly reported in both human and animal models of disease [22-26]. Discordant intensity distribution of AQP4 and GFAP was also observed in this study (Figure 5). Increased AQP4 immunolabelling in CM was not simply a consequence of astrocyte hypertrophy or hyperplasia since GFAP levels did not differ between CM and non-CM cases (Figure 4G). AQP4 did not appear to be enhanced around areas of fibrinogen leakage (Figure 4). There was no correlation in this study between the prevalence of any of the patterns of histological oedema and the degree of AQP-4 staining in a particular section. There were also no correlations with clinical, biochemical or histological parameters of severe malaria infection. There were no statistical associations with sequestration or microvascular congestion in the brain, or brain weight at the time of death.

## Discussion

Cerebral swelling is a common feature of CM in adults and children in radiological studies [8-12]. The degree of swelling varies, and the radiological features usually suggest that it results from excess intracranial blood volume. Intracranial pressures are often elevated in children with CM but not in adults [27,28]. Not all cerebral swelling seen radiologically or macroscopically at autopsy results from parenchymal cerebral oedema. Radiological evidence of frank oedema is less common although it is well described, particularly in fatal cases near to death. There are two main types of oedema recognized in the brain [29]. Vasogenic oedema is characterized by leakage of water into the perivascular and parenchymal extracellular compartments resulting from BBB breakdown. Cytotoxic oedema represents cellular swelling resulting from the breakdown of the normal membrane controls of fluid and ion transport that maintain osmotic homeostasis in neurones and other cells such as astroglia [30].

In this autopsy study, histological examination showed that microscopic oedema was a common finding in the post-mortem brains of fatal Vietnamese adults who died of severe malaria cases, but that brain weights were not significantly increased and that evidence for microscopic cerebral oedema was not associated with coma pre-mortem.

There are many reasons for the brain to be swollen in severe malaria. Seizures are often multiple and protracted (particularly in children) and there may be metabolic alterations because of liver dysfunction or renal failure (predominantly in adults). In acute cerebral malaria there is marked intravascular congestion of parasitized erythrocytes and uninfected erythrocytes resulting from sequestration, which increases the intravascular blood volume and therefore brain volume without altering overall cerebral blood flow. Apart from the disease process itself, treatment may result in excess fluid administration, or mechanical ventilation may undercompensate for the hypocapnia that accompanies metabolic acidosis and cause cerebral vasoconstriction.

Cerebral oedema can be variable and focal, around defined larger areas of white matter damage, so may not make a significant contribution to the overall brain water content ('volume' or 'weight') but might still have a negative impact on neuronal function. The host reaction to PRBC sequestration may differ in young patients with less multiorgan disease, perhaps because of relative functional immaturity of the BBB making oedema more likely in younger patients. In vivo studies of BBB function through examination of CSF protein partitioning support a greater degree of BBB breakdown in African children [31] compared to Vietnamese adults [32] although the degree of permeability and the intracranial pressure increases are much less than in pyogenic meningitis. A recent paper has examined the development of cerebral oedema in murine model of experimental cerebral malaria. This model has been known for some time to demonstrate increased vascular permeability associated with fatal outcome in *Plasmodium berghei*-infected mice. The results of this study indicated a strong association between cerebral oedema and experimental cerebral malaria in mice [33], which was not found in adult human fatal malaria cases.

Because of these remaining uncertainties as to the role of brain oedema in the pathogenesis of coma in human CM, the prevalence and patterns of cerebral oedema in post-mortem brain from adult fatal malaria cases was examined, and related to factors which could influence the formation of oedema, including systemic complications of disease such as renal failure, immunohistochemical evidence of vascular integrity at the blood-brain barrier, and the expression of the water transport channel Aquaporin 4. Several distinct histological forms of oedema were identified and found to co-exist in the same tissue section. The categorization of four different patterns of histological oedema used in this study was based initially on the accepted neuropathological differences between cytotoxic oedema and vasogenic oedema [29]. Examination of this series showed recognizable separate patterns within these, such as bubbly parenchymal oedema rather than pericellular cytotoxic oedema, or deposition of granular proteinaceous material rather than just perivascular clearing in vasogenic oedema. These have not been previously described and as such the classification of the neuropathological features of oedema described here contains novel classes, which may be inter-related.

The prevalence of loss of vascular integrity was heterogeneous within patient groups. Using both post-mortem and in vivo studies of CSF previous studies had demonstrated disruption of BBB function, but in a mild and reversible form that is not as marked as other inflammatory and infective encephalopathies [32]. In the current study, there were no significant differences in the prevalence of oedema or haemorrhages, or loss of vascular integrity, between CM and fatal malaria cases without neurological complications. Therefore there was not a simple relationship between BBB disruption, fibrinogen leakage, development of oedema and subsequent coma.

However, there were differences in oedema formation and patterns of fibrinogen leakage between different brain regions. In general, there was a greater degree of oedema in brainstem compared with cortex and to a lesser extent with diencephalon. For example, evidence of oedema between axonal fibre tracts could be observed in all brainstem sections but was observed in less than 30% of cortical sections from severe malaria cases. Extravasation of proteinaceous material into the perivascular Virchow-Robbins space in the cortex was more common in patients with longer survival times after admission, suggesting that these changes need time to develop in order to be observed pathologically. However, these correlations were not observed in the brainstem despite a high incidence of perivascular protein leakage.

Immunohistochemical analysis showed that fibrinogen leakage in the cortex was frequently diffuse around small vessels whereas the brainstem displayed fibrinogen leakage around both small and larger vessels in a cell-associated or diffuse pattern. Correlations between patterns of fibrinogen leakage and markers of organ dysfunction were observed in the brainstem but not in the cortex or diencephalon. These results can be interpreted in several ways. The brainstem may be more susceptible to vascular damage or parenchymal cell injury in severe malaria cases that have a fatal outcome, or perhaps different mechanisms of cell damage could be in operation resulting from differences in tissue architecture. The lack of difference between CM and non-CM cases suggests the observed loss of vascular integrity is unlikely to be the cause of cerebral symptoms in CM per se.

The VEGF pathway has been demonstrated to increase BBB permeability, so the correlation between evidence of VEGF signalling and evidence of BBB leakage including fibrinogen leakage or prevalence of histological types of oedema was examined. However, there were no correlations with pKDR immunolabelling. Despite this, involvement of the VEGF pathway in the modulation of vascular integrity during severe malaria cannot be completely ruled out, since the peak of VEGF signalling may have occurred before treatment or death, and the parasite itself may modulate VEGF signalling in severe malaria [34]. Other potential vasomodulatory pathways have been examined in the severe malaria, such as tumour necrosis factor, angiopoetin [35,36], erythropoietin and nitric oxide. Inflammatory processes within the brain may also synergize VEGF expression, given that CD-8 positive T-cells can induce BBB leakage due to neuronal VEGF expression in neuroinflammatory conditions [37].

In contrast with VEGF, a statistically nonsignificant trend was identified for AQP4 staining to be higher in the brainstem of CM cases compared with non-CM cases (*P *= .02). Brain injury can variably up- or down-regulate AQP4 expression depending on the disease studied or the experimental model used, in the face of retained GFAP expression [38]. AQP4 induction may have different roles at different time points in disease. Since AQP4 controls water fluxes into and out of the brain parenchyma it remains difficult to determine whether the increased expression contributes to oedema formation or is a sign of neuroprotective attempts at resolution. In this study, there was no correlation between AQP4 expression levels and the loss of vascular integrity in the severe malaria sections. However, previous studies have shown that enhanced AQP4 immunoreactivity can be detected around ischaemic foci even after brain oedema had resolved [39]. One possible scenario in severe malaria is that AQP4 does not prevent opening of the BBB or vasogenic oedema formation but, rather, promotes water clearance from the brain, limiting the development of cytotoxic oedema [40]. This would normally develop following extensive microvascular obstruction, similar to that directly observed in retina and rectal mucosa of CM patients [41,42]. Gene knockout studies in mice suggest multiple roles for AQP4 in regulation of water transport, astrocyte signalling and direct modulation of neuronal excitability [43,44]. In this study, AQP4 labelled processes were found around neuronal somata. Increased seizure duration and slowed potassium kinetics occur in mice lacking AQP4 channels [45]. This has been attributed to subcellular co-localisation of AQP4 with the inwardly rectifying potassium channel Kir 4.1, suggesting that AQP4 may participate in the coupled influx of water and K^+ ^that occurs after neural activity. The frequent occurrence of seizures as part of the clinical spectrum of CM may influence the pattern of AQP-4 expression.

This study was designed to determine any association between brain oedema and the neurological complications of severe malaria, in order to validate approaches to potential trials of neuroprotective adjuvant treatment. Autopsy based studies clearly have disadvantages in following the temporal sequence of cerebral swelling in CM. However planned studies differentiating between cytotoxic and vasogenic oedema on the basis of diffusion weighted functional MRI imaging should allow some comparison of these post-mortem findings with malaria patients *in vivo *[46]. Functional MRI imaging offers a promising way to study the effects of oedema on metabolic and pathological changes in both human CM and relevant animal models of the disease [47]

Recent treatment studies in both African children [28] and Indian adults [48] have confirmed that although cerebral swelling and associated increases in intracranial pressure often occur in paediatric cases of CM, treatment with mannitol, which reverses vasogenic oedema, is ineffective or harmful. Steroids, which also reduce oedema as well as inflammation, are also ineffective in adult CM [49]. Therefore, the processes by which malaria cause brain swelling and the relationship of this swelling to extravascular oedema are not straightforward, and may not offer the potential for adjuvant neuroprotective therapy.

## Conclusions

Brain swelling is a variable feature of cerebral malaria in adults, which is not accompanied by increased brain weight at autopsy or consistently elevated CSF opening pressures in all patients presenting with coma. Histological evidence of vasogenic oedema, representing changes to blood-brain barrier permeability, was common in fatal cases of both cerebral and non-cerebral malaria. Extravascular oedema may compound the increased intravascular volume resulting from sequestration and congestion. This study found different microscopic patterns of oedema, but no evidence that these correlated with pre-mortem coma, or the expression of molecular pathways involved in regulation of water transport into the brain. This implies that although brain swelling may contribute to neurological dysfunction in individual cases, cerebral oedema can be seen in non-comatose patients as well as cerebral malaria cases, and appears to be a consequence of malaria infection itself rather than being the specific cause of coma in cerebral malaria.

## Competing interests

The authors declare that they have no competing interests.

## Authors' contributions

IM and NS performed immunohistochemistry and image analysis, performed statistical analysis and helped draft the manuscript. NTHM and TTH collected post-mortem samples and clinical data. AD and EP participated in the design and coordination of the study and drafting the manuscript. GT, ND and NW conceived of the study, participated in its design and coordination and helped draft the manuscript. All authors read and approved the final manuscript.
